# 3D-Printed PEG–PLA/Gelatin Hydrogel: Characterization toward In Vitro Chondrocyte Redifferentiation

**DOI:** 10.1021/acsbiomaterials.4c02409

**Published:** 2025-03-20

**Authors:** Pacharapan Sonthithai, Pakkanun Kaewkong, Somruethai Channasanon, Siriporn Tanodekaew

**Affiliations:** National Science and Technology Development Agency (NSTDA), 111 Thailand Science Park, Phahonyothin Road, Klong Nueng, Klong Luang, Pathum Thani 12120, Thailand

**Keywords:** gelatin, polylactide, 3D printing, hydrogel, chondrogenic redifferentiation

## Abstract

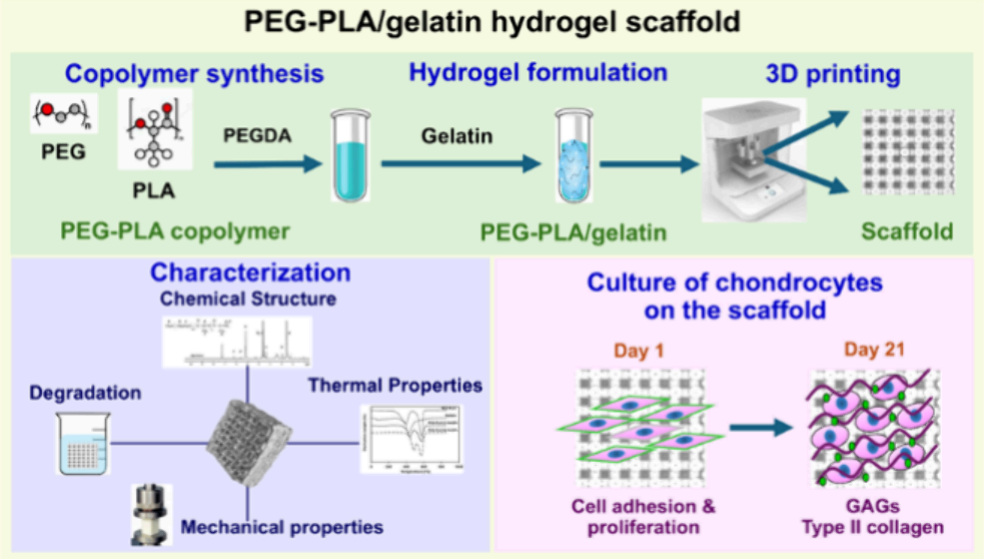

The advancement of 3D printing technology offers a sophisticated solution for tissue engineering and regenerative medicine. Several printable hydrogels have been developed with specific designs for certain tissues. However, there are few effective 3D-printed hydrogels for cartilage tissue engineering due to challenges with the hydrogel printability and the redifferentiation capacity of the articular chondrocytes on the hydrogel. This research study combined a PEG–PLA copolymer with gelatin to develop 3D-printed scaffolds for cartilage regeneration. Different hydrogel samples were prepared and studied regarding the effects of PLA chain length, gelatin content, and cross-linker concentration on the mechanical properties, swelling ability, and degradability of the hydrogels. An increase in the swelling ratio was observed, resulting in diminished compressive properties and accelerated degradation of the hydrogels with increased gelatin or decreased cross-linker and PLA chain length. Porcine articular chondrocytes were seeded onto the hydrogel scaffolds to assess cell adhesion, proliferation, and redifferentiation capability. Hydrogels with high swelling ability promoted the initial adhesion of cells on the scaffold, hence significantly increasing chondrocyte proliferation within 2 weeks of culture. Lowering the compressive modulus by increasing gelatin content improved chondrogenic redifferentiation. Glycosaminoglycan secretion was significantly enhanced when cells grew on hydrogels with greater amounts of gelatin. Furthermore, immunofluorescence staining of the cell-loaded hydrogels showed clusters of cells with a dense accumulation of a type II collagen network, a basis component of the cartilaginous matrix. Neither the PLA chain length nor the cross-linker amount affected chondrogenic function. The present study demonstrates that the PEG–PLA/gelatin hydrogels with increasing amounts of gelatin provide an optimal combination of swelling ratio, compressive modulus, and degradation rate, resulting in an appropriate environment to support the growth and redifferentiation of articular chondrocytes. This 3D-printed PEG–PLA/gelatin hydrogel will be useful for cartilage tissue engineering and possibly contribute to a new approach for cartilage defect treatment.

## 1 Introduction

Hydrogels can hold massive amounts of water while possessing mechanical properties that resemble the natural extracellular matrix, making them attractive for tissue engineering.^1^ The development of hydrogels, particularly in the field of tissue-engineered cartilage, is of interest due to the need for high-quality cartilage repair to address the limited self-repair capabilities of cartilage. In recent years, 3D printing technology has been introduced to create porous scaffolds. Several studies have reported the successful printing of hydrogel-based scaffolds with precise control of their properties through their shape and architecture. Different materials for hydrogel scaffolds have been developed including alginate, hyaluronic acid, collagen, and gelatin.^2−7^ However, the choice of hydrogel for cartilage tissue engineering is limited, which restricts the advancement of 3D-printed hydrogels for cartilage repair.

Articular cartilage injuries are a common clinical problem that affect billions of people worldwide and can lead to the deterioration of joints if left untreated. Current treatment strategies are classified as palliation, defect repair, cartilage restoration, and total knee replacement. Regenerative therapy by using scaffold and scaffold-free cells has been introduced recently as an alternative option for cartilage defect treatment.^8^ Gelatin methacrylate (GelMA) is one of the materials that has received extensive attention as a hydrogel-based scaffold for cartilage tissue repair. The tunable mechanical strength of GelMA hydrogel to mimic natural cartilage is crucial for chondrogenesis. The stiffness of GelMA hydrogel depends upon the degree of substitution of GelMA hydrogels, and higher stiffness allows for maintaining the chondrocyte phenotype.^9^ The microporous structures of GelMA hydrogel also influence chondrogenesis.^10^ Not only mechanical but also chemical properties of hydrogels play an important role in controlling cell functions. There is a rising number of chemically modified gelatin to gain additional cell functions, e.g., to direct the migration, growth, and organization of cells during tissue regeneration. GelMA incorporated with hyaluronic acid significantly enhanced chondrogenesis and facilitated matrix distribution, with corresponding improvements in mechanical properties. Likewise, the incorporation of chondroitin sulfate enhanced some aspects of chondrocyte differentiation, but to a lesser extent.^11^ The development of hydrogels with respect to their physical and chemical properties to manipulate the interaction between cells and scaffolds in such a way that directs cells toward a chondrogenic phenotype and promotes new matrix formation is a challenge for the successful application of hydrogels in cartilage regeneration.

Polylactide (PLA) is a synthetic biomaterial approved for in vivo applications that offers scaffold fabrication by 3D printing methods.^12,13^ PLA microspheres-embedded scaffolds containing decellularized cartilage matrix induced the expression of biochemical markers of cartilage development.^14^ PLA-based scaffolds with proper geometry exhibited excellent cell survival and improved the chondrogenic properties of the cells.^15^ In our previous work, PLA-based scaffolds were studied for bone scaffolding, and the incorporation of hydroxyapatite demonstrated great osteoinductivity, as evidenced by high levels of alkaline phosphatase activity and calcium deposition.^16,17^ To serve as scaffolds for cartilage, poly(ethylene glycol) (PEG) was intentionally introduced as a hydrophilic part into the hydrophobic PLA to enhance the potential of cartilage repair. Furthermore, gelatin was combined to favor cell behaviors such as cell attachment, proliferation, and differentiation. These hydrogel components have never been prepared for extrusion-based 3D printing and used as a substrate for chondrocyte culture. Gelatin, a natural extracellular matrix (ECM) protein derived from collagen, is expected to support cell viability and promote cartilaginous tissue formation, similar to findings with articular cartilage ECM methacrylate and collagen-based bioinks.^18,19^ Injectable, in situ-forming PEG–PLA hydrogels have been shown to serve as protein-friendly carriers for controlled delivery.^20^ The combination of the biological reactivity of gelatin with PEG–PLA expectedly provides a hydrogel that closely mimics native articular cartilage. Moreover, its physical and mechanical properties, as well as its degradation rate, can be tailored through the PEG–PLA ratio and an additional cross-linker to support tissue regeneration.

In this study, the composition of the PEG–PLA/gelatin hydrogel was optimized to enable extrusion-based 3D printing by the technique of direct ink writing. The PEG–PLA copolymers with varying PLA lengths were synthesized to prepare PEG–PLA/gelatin hydrogels. NMR and FTIR were studied to analyze the chemical structure, and TGA was performed to examine thermal stability. Swelling ratio, compression, and degradation tests were carried out to study the effects of PLA chain length, gelatin content, and cross-linker concentration on the physical and mechanical properties of the hydrogels. The potential application of PEG–PLA/gelatin hydrogels as scaffolds for cartilage tissue engineering was investigated by a cell proliferation assay. In addition, glycosaminoglycan (GAG) and type II collagen were measured to assess cartilage regeneration activity.

## 2 Materials and Methods

### 2.1 Materials

l-lactide was purchased from Purac Asia Pacific Pte. Ltd. Poly(ethylene glycol) methyl ether (*M*_n_ ∼ 2000) and poly(ethylene glycol) diacrylate (PEGDA, *M*_n_ ∼ 575) were purchased from Aldrich chemistry. Methacrylic anhydride was obtained from Sigma-Aldrich. Gelatin (type B) was received from Himedia LabChemicals & BioChemicals. All chemicals were used without purification.

### 2.2 Synthesis of Poly(ethylene glycol)- Polylactide Copolymers End-Capped with Methacrylate Group (PEG–PLA)

The synthesis of PEG–PLA proceeded following a two-step reaction, as previously reported.^13^ Briefly, the alkoxide of poly(ethylene glycol) methyl ether was freshly prepared to initiate the anionic ring-opening polymerization of l-lactide, which was subsequently end-capped by reacting with methacrylic anhydride. The molecular weight and polydispersity index (PDI) of the PEG–PLA copolymers with different PLA block lengths were characterized using GPC (Waters e2695, Waters Corporation). The molecular weights (PDI) of PEG–PLA1, PEG–PLA2, and PEG–PLA3 were found to be 8390 (1.5), 12,200 (1.7), and 13,810 (1.5) g/mol, respectively.

Their chemical structure was studied by ^1^H NMR spectroscopy (Bruker DPX-300 spectrometer) and FTIR spectroscopy (Spectrum spotlight300, PerkinElmer) in micro attenuated total reflectance (Micro-ATR) mode.

### 2.3 Preparation of PEG–PLA/Gelatin Hydrogel Scaffolds

**Figure 1:**
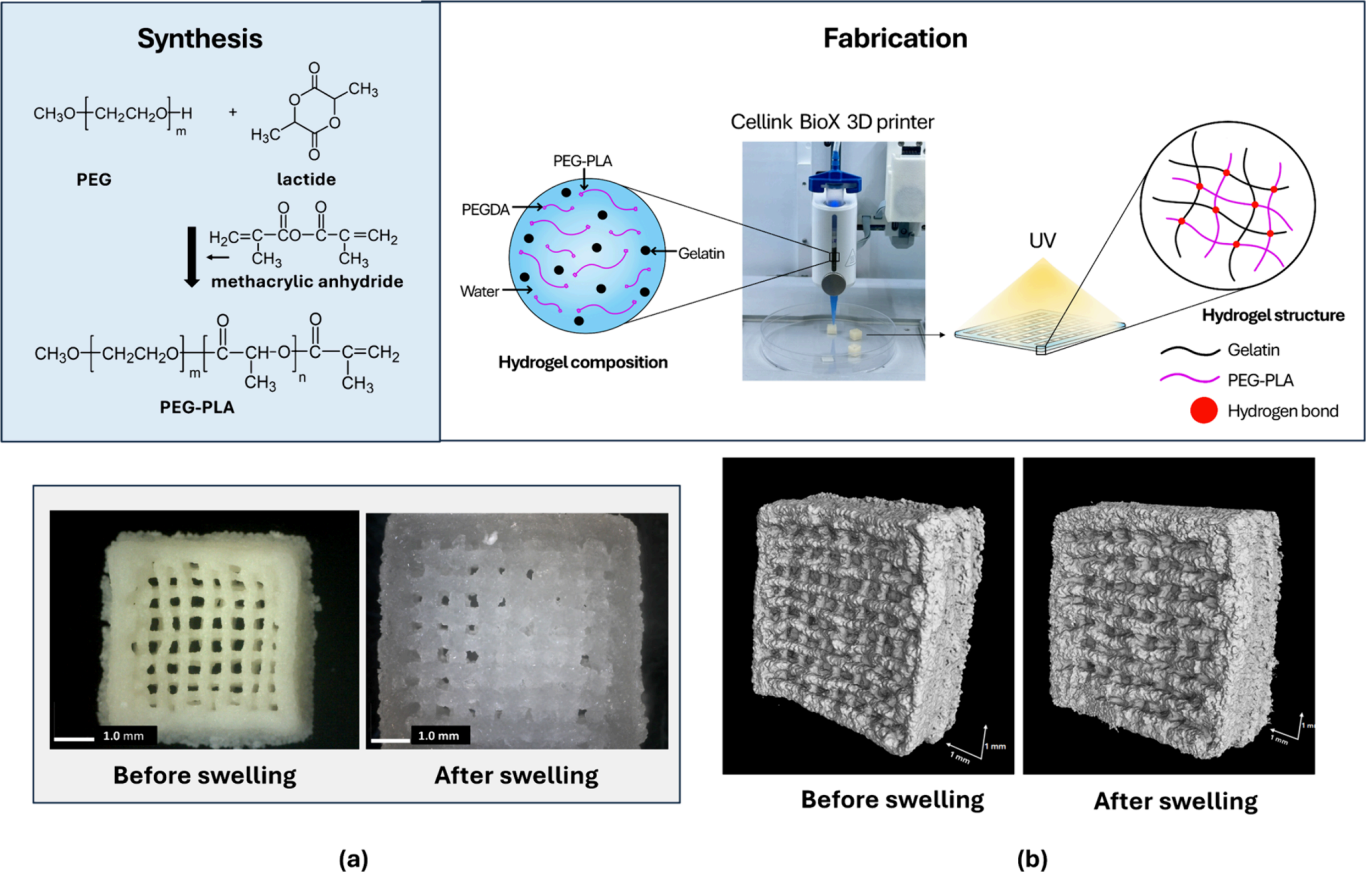
Preparation of PEG–PLA/gelatin hydrogel scaffolds and images of 3D-printed sample (PEG–PLA2(1-Gel)9%) before and after swelling taken with a Dino-Lite microscope (DinoCapture 2.0 software, version 1.5.51) (a) and X-ray computed tomography (X-ray CT, Phoenix V/tome/x M240, Waygate technologies) (b).

Gelatin was precross-linked using the carbodiimide coupling reaction and subsequently crushed into fine particles (50–150 μm). The PEG–PLA copolymer was then mixed with gelatin particles, PEGDA (a cross-linker), Irgacure 2959 (a photoinitiator), and water by using a planetary mixer/deaerator (Mazerustar KK-250s, Kurabo Industries Ltd.). The amounts of PEG–PLA, gelatin, and PEGDA were varied corresponding to the hydrogel compositions shown in Table 1, where X represents the weight ratio of gelatin to PEG–PLA copolymer, and Y indicates the weight percentage of PEGDA. The PEG–PLA copolymer used in each formulation was labeled based on the PLA length, namely, PEG–PLA1, PEG–PLA2, or PEG–PLA3. For example, PEG–PLA1(1-Gel)9% contains 9.1% PEGDA, with a weight ratio of gelatin to PEG–PLA1 of 1:1, each comprising 14.5 wt %. Hydrogels were fabricated into scaffolds using direct ink writing (Bio X, Cellink), an extrusion-based additive manufacturing method. They were extruded through a nozzle (diameter, 400 or 250 μm) with controlled pressure (75–180 kPa) and printing speed (1–4 mm s^–1^), then exposed to UV radiation to radically initiate polymerization reactions, as illustrated in Figure 1.

**Table 1:** Hydrogel Composition

	hydrogel composition (wt %)			
PEG–PLA(X-Gel)Y%	PEG–PLA	gelatin	PEGDA	water
PEG–PLA(0-Gel)9%	36.4	0	9.1	54.5
PEG–PLA(1-Gel)9%	14.5	14.5	9.1	61.9
PEG–PLA(1-Gel)13%	13.9	13.9	13.0	59.2
PEG–PLA(2-Gel) 9%	11.4	22.7	9.1	56.8
PEG–PLA(3-Gel) 9%	8.7	26.0	9.1	56.2

### 2.4 Thermal Characterization

A thermogravimetric analyzer (TGA, TGA/SDTA851e, Mettler-Toledo International Inc.) was used to observe the thermal properties of hydrogels at a heating rate of 10 °C/min under a nitrogen atmosphere from room temperature to 600 °C.

### 2.5 Swelling Ratio

The swelling ratio was evaluated by the weighing method. The hydrogel samples (6 mm in diameter and 1 mm in height) were swollen in deionized water at room temperature for 3 h to obtain equilibrium swelling. The samples were then removed, blotted with tissue paper, and weighed (*W*_s_). The swollen samples were dried in an oven at 37 °C to constant weight and weighed again (*W*_d_). The swelling ratio was calculated by the following formula:
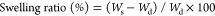
where *W*_s_ and *W*_d_ are the weight of the swollen and dried hydrogel samples, respectively.

### 2.6 Compressive Testing

The compressive properties of the hydrogels (*n* = 5) were measured using a universal testing machine (model: 5943, Instron Corp). The hydrogel samples (4 mm diameter and 6 mm height) were swollen in deionized water at room temperature for 3 h prior to the test. The compressive stress–strain curve was obtained by loading 100 N at a rate of 1 mm/min. The compressive modulus (MPa) was calculated from the stress–strain curve as the slope of the curve within the strain range of 5 to 10%. The compressive strength (CS) was calculated as follows:
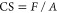
where *F* denotes the maximum load at break in Newton, and *A* denotes the area of the specimen on compression in square millimeters.

### 2.7 Degradation

The degradation degree was determined by measuring the weight loss of the hydrogel after incubation in PBS (pH7.4) at 37 °C. The hydrogel samples (6 mm diameter and 1 mm height) were dried and weighed (*W*_i_) before being immersed in PBS, with the PBS refreshed every week. After 2 and 4 weeks, the swollen hydrogels were taken out, dried, and weighed (*W*_*t*_). The change in weight of the hydrogels was calculated as follows:
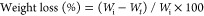
where *W*_i_ and *W_t_* are the weight of the dry hydrogels at initial and time *t*, respectively.

### 2.8 In Vitro Chondrocyte Culture

The printed hydrogel samples (6 mm diameter and 1 mm height) were sterilized with 75% ethanol and air-dried in a laminar flow prior to the test. Each biological test was performed in triplicate (*n* = 3).

#### 2.8.1 Cell Isolation and Culture

The experimental protocol was conducted with approval from the IACUC of NSTDA (COA-014-2567). Porcine articular chondrocytes were isolated from the knee joints of a newborn pig. The cartilage sections were sliced and minced into pieces. After washing with phosphate-buffered saline (PBS), the pieces of cartilage were digested with 0.2% (w/v) collagenase (Sigma-Aldrich, St. Louis, MO) in Dulbecco’s modified Eagle’s medium (DMEM; Gibco, Grand Island, NY) containing 1% antibiotic-antimycotic solution (Gibco) for 6 h at 37 °C. Cells were collected by centrifugation at 1500 rpm for 10 min. The cell pellet was resuspended in culture medium consisting of DMEM medium containing 10% (v/v) fetal bovine serum (FBS; Gibco) and 1% antibiotic-antimycotic solution. The Isolated chondrocytes were subsequently incubated in a humidified atmosphere of 5% CO_2_ at 37 °C. When the primary cultures reached 80% confluence, the chondrocytes were harvested using trypsin (Gibco). The cells were expanded and harvested at the third passage for seeding on hydrogels.

#### 2.8.2 Cell Adhesion and Proliferation

Cell adhesion and proliferation were determined by the MTT assay. The hydrogels were placed in a 48-well plate and soaked in a serum-free medium overnight before seeding with primary chondrocytes at a density of 4 × 10^4^ cells/mL/hydrogel. After overnight incubation to allow for the attachment of cells, the cell-seeded hydrogels were moved to a new 48-well plate and cultured in DMEM containing 10% (v/v) FBS and 1% antibiotic-antimycotic solution. The medium was changed every other day. The MTT solution (0.5 mg/mL) was added to the samples at day 1 of culture to determine cell adhesion on the hydrogels and at days 7, 14, and 21 to assess cell proliferation throughout the experiment. After the incubation of the samples in MTT solution for 4 h, the resultant formazan crystals were solubilized with 500 μL of dimethyl sulfoxide (DMSO). A 200-μL aliquot of the formazan suspension was transferred to a new well, and the absorbance was measured at 570 nm using a microplate reader (VICTORTMX4 multilabel plate reader, PerkinElmer). Hydrogels without cells were included in the assay for subtracting the background spectrum in different hydrogel formulas. The surface area of hydrogel scaffolds was measured using DinoCapture 2.0 software (version 1.5.51). At each point, the absorbance value of viable cells on the hydrogel was divided by its corresponding surface area and calculated for cell viability. The proliferation of chondrocytes on PEG–PLA1(1-Gel)9% at day1 was set as 100%.

#### 2.8.3 Determination of GAG Accumulation

Total GAG content was quantified by a colorimetric dimethylmethylene blue (DMMB) assay. Briefly, the cell-seeded hydrogels at day 21 were lyophilized prior to recording their dry weight. The dried samples were subsequently digested at 60 °C for 18 h in a papain buffer (15 U/mL papain (Sigma, USA), 5 mM l-cysteine, 10 mM Na_2_HPO_4_, 5 mM EDTA, pH 7.5). A 100-μL aliquot of the papain-digested extract was mixed with an equal volume of DMMB dye (Sigma, USA). The absorbance was measured at 525 nm using a microplate reader. The concentration of GAGs was extrapolated using a standard curve of chondroitin-6-sulfate (Sigma, USA) and individually normalized to the dry weight. The amount of GAG accumulation is expressed as ng per mg dry weight of the scaffold.

#### 2.8.4 Immunofluorescence Staining for Type II Collagen

The chondrocytes cultured on hydrogels at day 21 were fixed in 4% paraformaldehyde for 15 min at room temperature, washed with PBS, and permeabilized in 0.1% Triton X-100/PBS for 10 min. For blocking nonspecific antibody bindings, samples were immersed in 5% BSA/PBS for 16 h at 4 °C prior to specific staining with antitype II collagen antibody (1:100, MAB1330, Millipore, USA) at 4 °C overnight. The samples were washed twice with PBS and incubated with an Alexa Flour647 goat antimouse secondary antibody (1:200, A2124, Life Technologies, USA) for 2 h at room temperature. To stain F-actin filaments and the nucleus of the chondrocytes, the samples were further incubated in FITC-phalloidin (1:500, Life Technologies, USA) for 45 min and DAPI (1:1000, Life Technologies, USA) for 2 min, respectively. The fluorescently stained samples were visualized and imaged using a confocal laser scanning microscope (FV10i, Olympus, Japan).

### 2.9 Statistical Analysis

Data are presented as mean ± SD. Statistical analyses were performed using SPSS program version 19.0 (SPSS, Inc., Chicago, IL) with one-way ANOVA followed by Duncan’s multiple range test. The differences between sample means were considered statistically significant and marked with different letters when the p-value was less than 0.05.

## 3 Results

### 3.1 Chemical Structure Analysis

**Figure 2:**
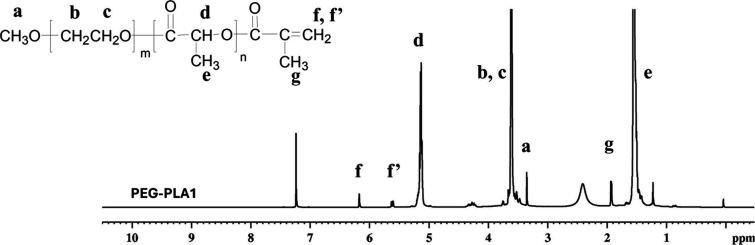
^1^H NMR spectrum of the PEG–PLA copolymer.

**Figure 3:**
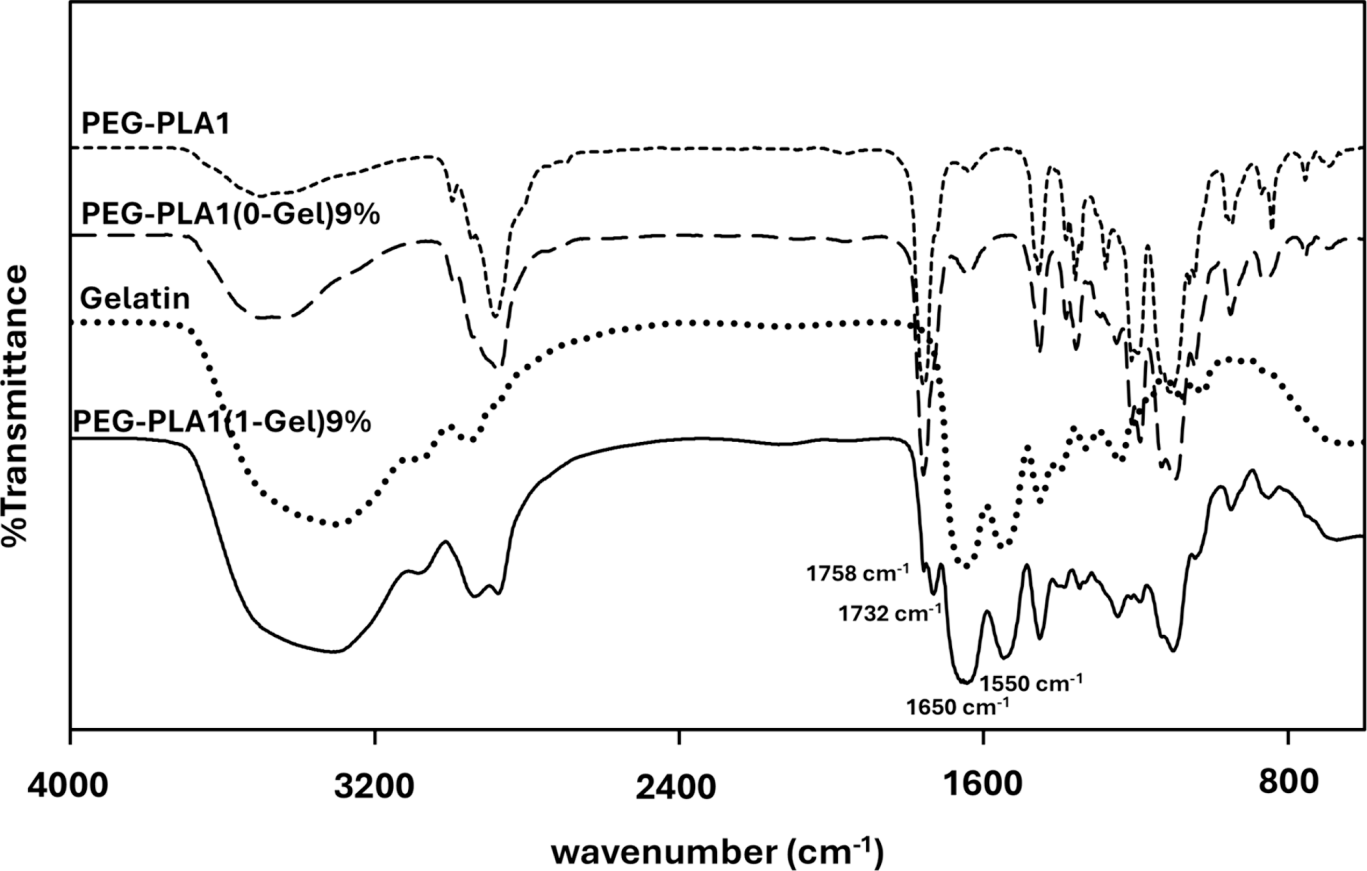
FTIR spectra of PEG–PLA copolymer, gelatin, and PEG–PLA/gelatin hydrogels.

The chemical structures of PEG–PLA determined by ^1^H NMR spectroscopy and FTIR are shown in Figures 2 and 3, respectively. From the ^1^H NMR spectrum, the −CH_2_O- protons of the PEG block were observed around 3.60 ppm, whereas the −CHO- and −CH_3_ protons of the PLA block appeared at 5.1 and 1.5 ppm, respectively. The characteristic peaks appearing at 5.6 and 6.2 ppm designated the end-capped methacrylate groups. The degree of substitution, i.e., the number of methacrylate groups per copolymer, was 0.51, 0.57, and 0.9 for PEG–PLA1, PEG–PLA2, and PEG–PLA3, respectively, as calculated from the proton signals of the methacrylate group and the lactide backbone.

The FTIR spectra of the PEG–PLA copolymer showed a characteristic absorption peak of the carbonyl groups at 1758 cm^–1^, whereas that of gelatin exhibited the characteristic amide absorption bands at 1650 and 1550 cm^–1^. The FTIR spectra of hydrogels with varying gelatin contents (PEG–PLA1(1-Gel)9%, PEG–PLA1(2-Gel)9%, and PEG–PLA1(3-Gel)9%) were similar, exhibiting a combination of the PEG–PLA and gelatin characteristic peaks, with a new peak appearing at 1732 cm^–1^. This peak, which was not present in the hydrogel without gelatin (PEG–PLA1(0-Gel)9%), indicated interactions between PEG–PLA and gelatin. The carbonyl groups of PEG–PLA formed hydrogen bonds with the hydrogen donor groups of gelatin, resulting in a shift of the free carbonyl peak at 1758 cm^–1^ to a lower wavenumber. This hydrogen-bonded carbonyl peak became more intense, suggesting greater bonding interactions at a higher gelatin amount (result not shown).

### 3.2 Thermal Property

**Figure 4:**
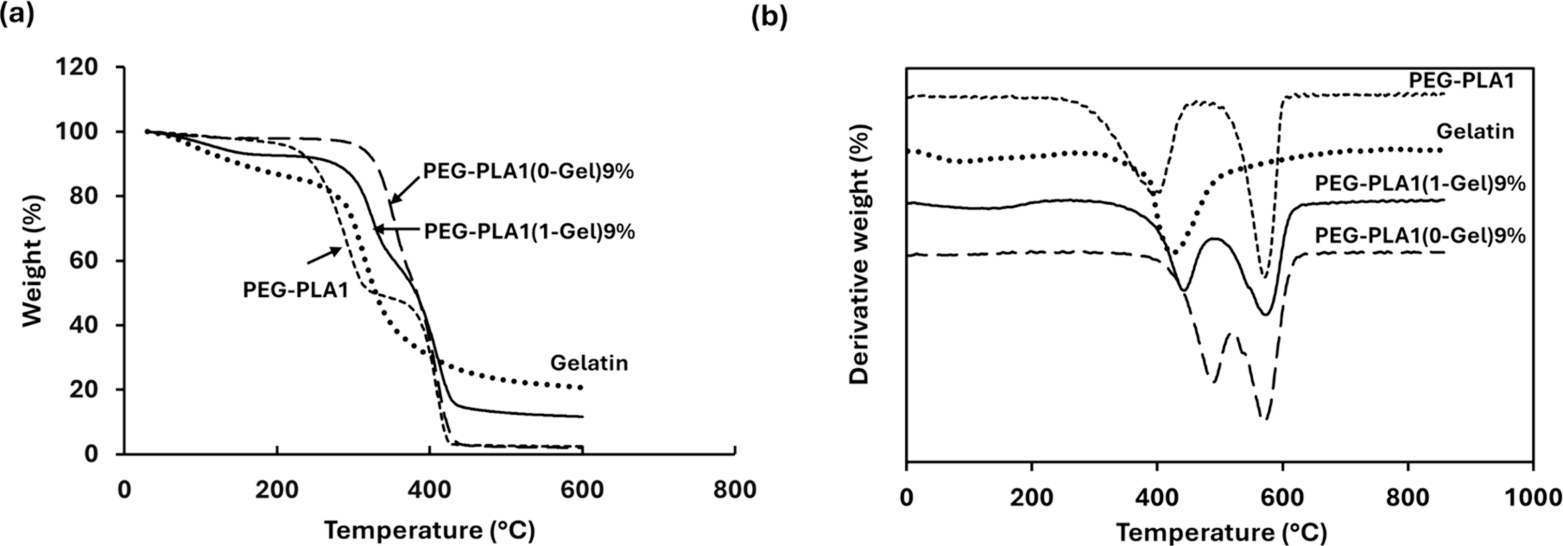
Thermogravimetric (a) and derivative curves (b) of PEG–PLA copolymer, gelatin, and PEG–PLA/gelatin hydrogels.

TGA results in Figure 4 confirmed the polymeric network forming of the hydrogel. The thermal decomposition of gelatin showed two steps of weight loss. The first one was in a temperature range of 40–200 °C, attributed to the loss of absorbed and bound water, and the second one was in a temperature range of 200–400 °C, involving protein chain decomposition. The PEG–PLA copolymer also experienced two loss stages: the first one in a temperature range of 190–350 °C, due to the thermal decomposition of the polymer chain, and the second one in a temperature range of 350–450 °C, due to carbon burning. The thermal decomposition of the hydrogel without gelatin (PEG–PLA1(0-Gel)9%) exhibited the best thermal stability, as expected for the thermal behavior of the cross-linked PEG–PLA polymer. The thermal decomposition of hydrogels with varying gelatin contents (PEG–PLA1(1-Gel)9%, PEG–PLA1(2-Gel)9%, and PEG–PLA1(3-Gel)9%) was similar, showing two decomposition peaks. These peaks shifted to lower temperatures than those of PEG–PLA1(0-Gel)9%, due to the incorporation of gelatin. However, their thermal stability remained superior to that of the original gelatin, indicating the formation of bonds between gelatin and PEG–PLA.

### 3.3 Swelling Ability

**Figure 5:**
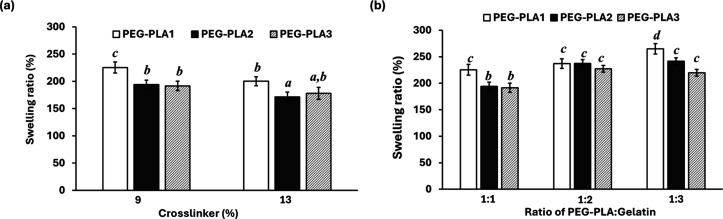
Swelling ratio of PEG–PLA/gelatin hydrogels: (a) different amounts of cross-linker at a 1:1 weight ratio of PEG–PLA: gelatin, and (b) different amounts of gelatin at 9% cross-linker. Different letters indicate statistically significant differences (*p* < 0.05).

The swelling ratio of hydrogels with varying hydrogel compositions is presented in Figure 5. The swelling degree of all the tested hydrogels increased rapidly and approached equilibrium at about two times of their original weight after 3 h (result not shown). The swelling ratio increased with a decreased amount of cross-linker, as expected (Figure 5a). The swelling ratio also increased when a higher amount of gelatin was introduced into the hydrogel (Figure 5b). The hydrogels derived from the PEG–PLA1 copolymer, with the shortest PLA chain length, showed significantly higher swelling ratios than the other hydrogels.

### 3.4 Compressive Property

**Figure 6:**
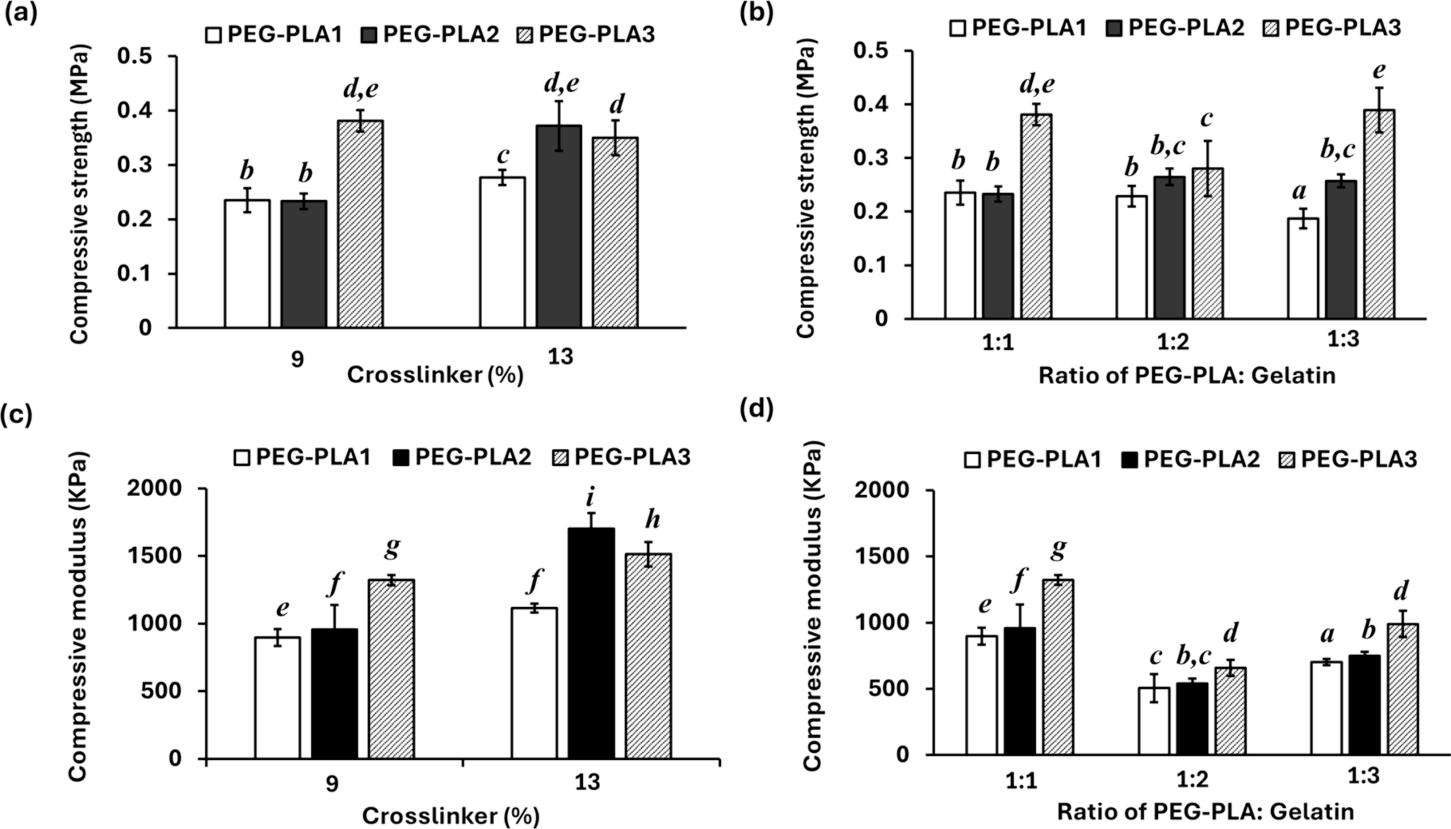
Compressive strength (a, b) and modulus (c, d) of PEG–PLA/gelatin hydrogels: (a, c) different amounts of cross-linker at a 1:1 weight ratio of PEG–PLA: gelatin and (b, d) different amounts of gelatin at 9% cross-linker. Different letters indicate statistically significant differences (*p* < 0.05).

Figure 6 shows the compressive strength and modulus of the hydrogels, which correspond well with the swelling findings. The less the swelling of the hydrogels, the higher the mechanical strength. Therefore, the hydrogels derived from the PEG–PLA3 copolymer, which exhibited the least swelling, demonstrated the highest compressive properties among the hydrogels.

### 3.5 Degradation

**Figure 7:**
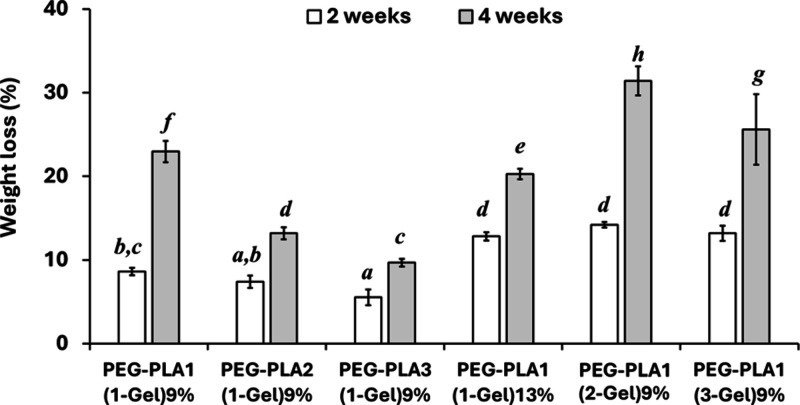
Weight loss of PEG–PLA/gelatin hydrogels against time.

Figure 7 shows the weight loss of PEG–PLA/gelatin hydrogels over degradation time. Hydrogels with longer PLA chains (PEG–PLA2(1-Gel)9% and PEG–PLA3(1-Gel)9%) exhibited a slower rate of degradation. The degradation rate appeared to be faster in hydrogels with higher gelatin amount (PEG–PLA1(2-Gel)9% and PEG–PLA1(3-Gel)9%).

### 3.6 Cell Adhesion and Proliferation

**Figure 8:**
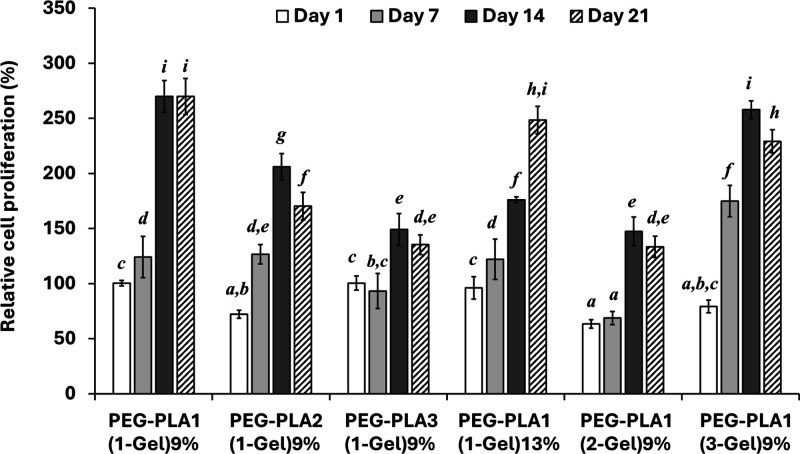
Proliferation of chondrocytes on 3D-printed hydrogels. Cell viability was assessed by MTT assay on 1, 7, 14, and 21 days of culture. The cell proliferation on PEG–PLA1(1-Gel)9% was set at 100% and those on the others were relatively compared. Different letters indicate statistically significant differences (*p* < 0.05).

Hydrogel samples were selected for biological studies to examine the effects of PLA chain length, gelatin content, and cross-linker concentration on cell adhesion and function. The effects of the hydrogel on cell adhesion and proliferation after chondrocyte culture for 1, 7, 14, and 21 days are shown in Figure 8. The cell proliferation at each time point on all hydrogels was presented as a percentage with respect to that of day 1 on PEG–PLA1(1-Gel)9%. Differences in the % cell viability were found among hydrogels with varying compositions. The chondrocytes adhered well to PEG–PLA1(1-Gel)9%), with slightly diminished adhesion as the gelatin content increased (PEG–PLA1(2-Gel)9% and PEG–PLA1(3-Gel)9%). Moreover, neither the increase in PLA chain length (PEG–PLA2(1-Gel)9% and (PEG–PLA3(1-Gel)9%) nor the amount of cross-linker (PEG–PLA1(1-Gel)13%) improved chondrocyte adhesion when compared to PEG–PLA1(1-Gel)9%.

In long-term culture, chondrocytes reached a maximum proliferation rate on day 14 on hydrogel scaffolds, except PEG–PLA1(1-Gel)13%, and could maintain their viability to the end of the culture on day 21. The chondrocytes showed a slow growth rate on the hydrogel with a high amount of cross-linker (PEG–PLA1(1-Gel)13%). A decline in cell proliferation on PEG–PLA2(1-Gel)9% and PEG–PLA3(1-Gel)9% was observed when compared with PEG–PLA1(1-Gel)9%, suggesting a negative effect of an increasing PLA chain length on cell proliferation. The hydrogel with the highest amount of gelatin (PEG–PLA1(3-Gel)9%) significantly enhanced chondrocyte proliferation on day 7, and cells continued to proliferate well until day 14; however, the proliferation slightly declined on day 21. The result indicated that the PEG–PLA1(1-Gel)9% hydrogel supported the proliferation of chondrocytes most effectively. The increase of PLA chain length, cross-linker, and gelatin did not improve cell proliferation.

### 3.7 Glycosaminoglycan (GAG) Accumulation

**Figure 9:**
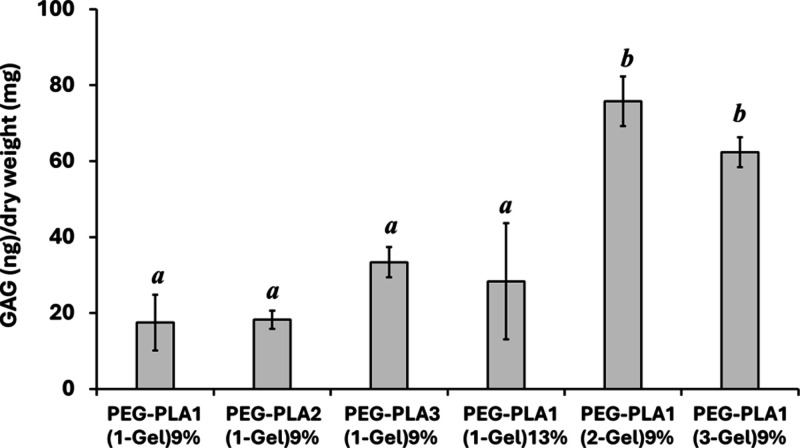
GAG accumulation in chondrocytes cultured on hydrogels on day 21. GAGs were quantified by DMMB and normalized to the dry weight of each hydrogel. Different letters indicate statistically significant differences (*p* < 0.05).

The chondrogenic function of cells cultured on each PEG–PLA/gelatin hydrogel was determined by measuring the production of cartilaginous ECM that are glycosaminoglycans (GAGs) and type II collagen. After 21 days of chondrocyte culture, the secretion level of GAGs was quantified and normalized to the dry weight of the individual hydrogels. There was no significant difference in the amount of GAG production between the chondrocytes cultured on hydrogels with varying PLA chain lengths (PEG–PLA1(1-Gel)9%, PEG–PLA2(1-Gel)9%, and PEG–PLA3(1-Gel)9%), and cross-linker concentration (PEG–PLA1(1-Gel)13%), as shown in Figure 9. Interestingly, chondrocytes grown on hydrogels with high gelatin content (PEG–PLA1(2-Gel)9% and PEG–PLA1(3-Gel)9%) showed a significantly higher level of GAG production, although their initial adhesion and proliferation rates on both scaffolds were slightly inferior to those cultured on the PEG–PLA1(1-Gel)9% hydrogel.

### 3.8 Type II Collagen Secretion

**Figure 10:**
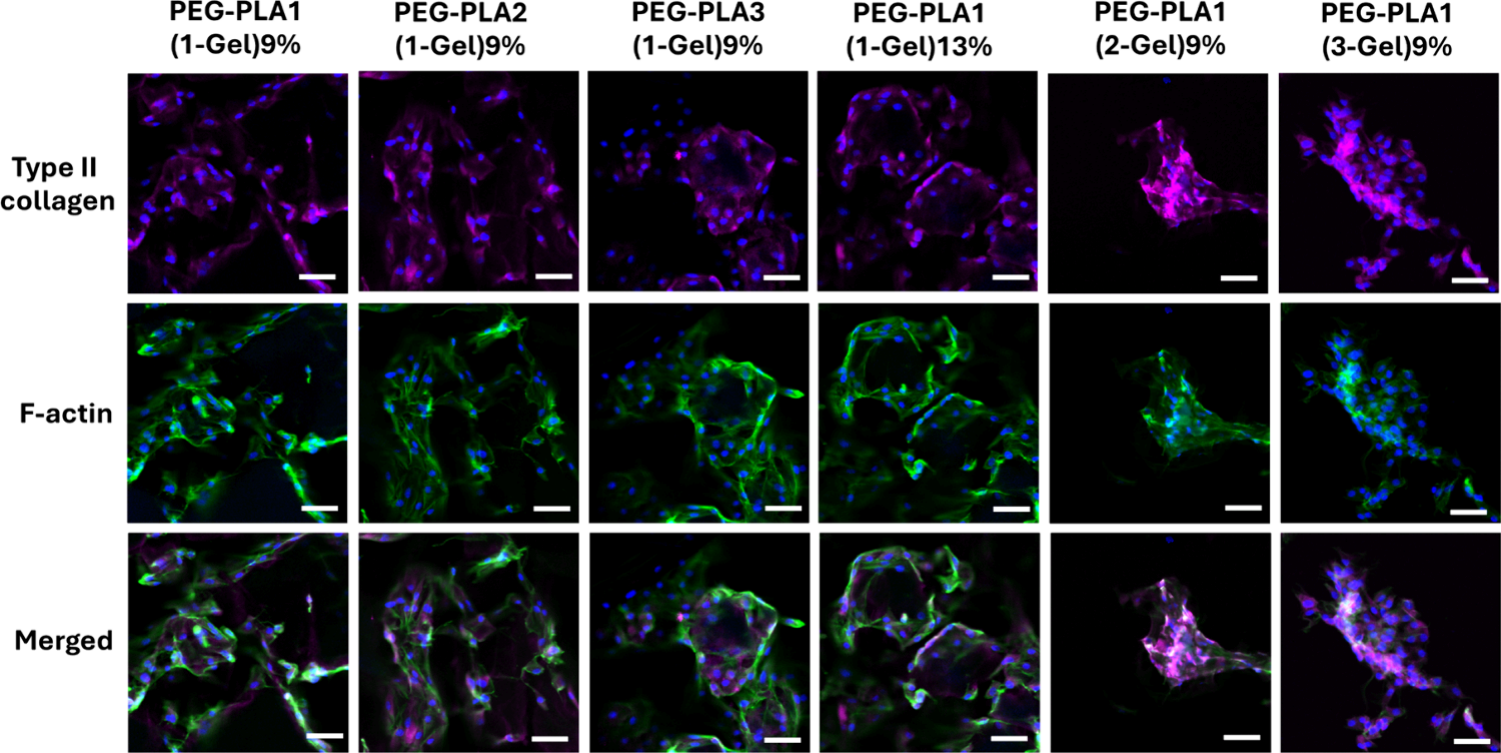
Secreted type II collagen from chondrocytes grown on hydrogels for 21 days. Immunofluorescence staining for type II collagen is shown in pink. Cell nuclei and F-actin were stained in blue with DAPI and green with FITC-phalloidin, respectively. Scale bars: 50 μm.

Despite the assessment of cell function by the level of GAG accumulation, type II collagen production from chondrocytes cultured on hydrogels was also detected by using immunofluorescence staining and confocal microscopy. As shown in Figure 10, all cell-seeded hydrogels displayed pink-stained type II collagen, suggesting the redifferentiation of chondrocytes on all hydrogels. The acquired images showed that cells formed clusters, rather than individual cells, with high-intently pink-stained type II collagen on PEG–PLA1(2-Gel)9% and PEG–PLA1(3-Gel)9%. Meanwhile, chondrocytes on the others, PEG–PLA1(1-Gel)9%, PEG–PLA2(1-Gel)9%, PEG–PLA3(1-Gel)9%, and PEG–PLA1(1-Gel)13%, localized as single cells that connected each other with a comparatively low intensity of pink fluorescence-stained type II collagen networks. The higher the fluorescence intensity of pink-stained type II collagen, the more the redifferentiation of chondrocytes on the scaffold. The F-actin filaments of chondrocytes were substantially stained on all hydrogel scaffolds, with more elongated structures observed in chondrocytes cultured on PEG–PLA1(1-Gel)9%, PEG–PLA2(1-Gel)9%, PEG–PLA3(1-Gel)9%, and PEG–PLA1(1-Gel)13%. Notably, some chondrocytes on these scaffolds showed a spindle-shaped morphology, which is a typical morphology of dedifferentiated cells. These cells tend to produce less type II collagen and aggrecan and instead produce more type I collagen. In contrast, the structural F-actin fibers were less elongated and were only partially visualized in chondrocytes cultured on PEG–PLA1(2-Gel)9% and PEG–PLA1(3-Gel)9%. The more type II collagen production, the fewer F-actin fibers were observed. The chondrocytes exhibiting a chondrogenic phenotype (round shape) were seen on these two scaffolds, indicating that the culture of chondrocytes on hydrogel scaffolds with a higher gelatin amount could lead to more effective production of ECM and a less fibroblast-like phenotype. During the process of chondrogenic redifferentiation, chondrocytes maintain a round or polygonal shape and produce the ECM of cartilage, including type II collagen and GAGs, which provide structural support and elasticity to cartilage. The localization of F-actin fibers also depicted cell clustering on PEG–PLA1(2-Gel)9% and PEG–PLA1(3-Gel)9%, similar to the arrangement of type II collagen fibers. The results on type II collagen production and F-actin arrangement were in good concordance with GAG accumulation, suggesting that the increase in the gelatin content in the PEG–PLA/gelatin hydrogels promoted the redifferentiation of chondrocytes.

## 4 Discussion

PEG–PLA copolymers with varying gelatin amounts were prepared to fabricate hydrogel scaffolds using the direct ink writing technique. The PEG–PLA/gelatin hydrogel possessed an interpenetrated network structure (IPN), resulting from UV-initiated radical polymerization of PEGDA. Increasing PEGDA content to 13 wt % yielded the hydrogel with a higher cross-linking density. In addition, there were intermolecular interactions with hydrogen bonds between the amino groups of gelatin and the carbonyl groups of PEG–PLA, as demonstrated by FTIR. The FTIR spectrum showed that not all carbonyl groups formed hydrogen bonds. The peak of free carbonyl at 1758 cm^–1^ coexisted with the peak of hydrogen-bonded carbonyl at 1732 cm^–1^. The number of carbonyl and amino groups was influenced by the PLA chain length and gelatin content, respectively, which determined the extent of intermolecular bonding and hence affected the hydrogel properties.

Generally, hydrogels with high water sorption are desirable for biomedical applications due to their benefits in permeability and biocompatibility. In this study, the swelling ratio of different hydrogels, which indicated water sorption capacity, was determined. The higher cross-linking density with increased PEGDA content resulted in a more compact IPN structure, and thus less swelling of the hydrogels. Regarding PLA chain length, PEG–PLA1 has a shorter PLA chain than PEG–PLA2 and PEG–PLA3, implying less hydrophobicity and thus better wetting ability. Furthermore, the small number of carbonyl groups from a short PLA chain resulted in a loose intermolecular network, which favored the water uptake of hydrogels. Consequently, the hydrogels derived from the PEG–PLA1 copolymer had higher swelling ratios than those hydrogels of PEG–PLA2 and PEG–PLA3. Regarding the gelatin amount, increasing the gelatin content in the hydrogel enhanced the number of amino groups available for bonding. This network formation was clearly evidenced from FTIR, which revealed a stronger hydrogen-bonded carbonyl peak with an increased gelatin amount. The dense network formation was opposed to water penetration; however, water absorption was found to be increased. This was likely due to the ability of gelatin to bind large amounts of water. As a result, it was observed that the swelling ratio increased as gelatin content increased.

The compressive properties of the hydrogels clearly depended on the swelling ability. The moduli of the PEG–PLA/gelatin hydrogels were in the range of 700–1800 KPa, which displayed interesting effects on chondrocyte culture, from cell attachment to redifferentiation. The initial critical phase of chondrogenesis is characterized by cell adhesion. Several studies have demonstrated that cell adhesion is strongly influenced by the hydrophilicity of surfaces.^21,22^ In this study, the PEG–PLA/gelatin hydrogels derived from the PEG–PLA1 copolymer, which has the shortest PLA chain length, showed good cell adhesion and proliferation. This enhanced performance correlated with their higher hydrophilicity, as measured by the swelling ratio. Likewise, the higher water sorption of the hydrogels with lower cross-linking density aligned with the proliferation results, which showed increased cell numbers of PEG–PLA1(1-Gel)9% hydrogels compared to PEG–PLA1(1-Gel)13% hydrogels on day 14. It was noted that the addition of gelatin enhanced hydrogel hydrophilicity; however, cell adhesion did not improve as expected. This was attributed to the mechanical properties of the hydrogels influencing cell responses. Previous studies have shown poor cell adhesion on scaffolds with low compressive moduli.^23,24^ The compressive modulus also affected chondrocyte functions, as reported by Choi et al. Their work on 3D-printed gelatin/hyaluronic acid scaffolds with varying cross-linker concentrations revealed that scaffolds with low compressive moduli enhanced chondrocyte differentiation.^25^ Similarly, Sarem et al. demonstrated that softer scaffolds promoted the formation of cartilaginous tissue.^26^ These findings were consistent with our study, which revealed that the hydrogels with lower compressive modulus, PEG–PLA1(2-Gel)9% and PEG–PLA1(3-Gel)9%, had higher levels of GAG accumulation compared to PEG–PLA1(1-Gel)9%. Accordingly, an enrichment of cartilaginous matrix was expressed on those hydrogels as cell clusters with a dense accumulation of type II collagen. On the other hand, PEG–PLA1(1-Gel)9%, despite exhibiting good cell adhesion and proliferation, showed dedifferentiated cells with reduced glycosaminoglycan secretion and type II collagen expression. The compressive modulus of native human articular cartilage varies by cartilage depth zone, which is ranging from 240 to 1000 kPa.^27,28^ The biological performance of the PEG–PLA/gelatin hydrogel scaffold was attributed not only to the chemical composition similarity between gelatin and the collagen matrix of articular cartilage^29,30^ but also to the compressive modulus, which is close to that of the native tissue, especially PEG–PLA1(2-Gel)9% and PEG–PLA1(3-Gel)9%.

Our study showed that the hydrophilicity, compressive modulus, and degradation properties of the hydrogels play important roles in chondrocyte response. Cell adhesion and proliferation, which are crucial during the initial stages of chondrogenesis, are strongly influenced by the hydrophilicity of materials. In contrast, chondrocyte functions are enhanced by a low compressive modulus as well as a degradation rate that aligns with tissue growth and regeneration. The hydrogels with increasing amounts of gelatin, PEG–PLA1(2-Gel)9% and PEG–PLA1(3-Gel)9%, achieved the optimal combination of swelling, stiffness, and degradation rate to support chondrocytes in maintaining their chondrogenic phenotype and promoting cartilage tissue regeneration. The hydrogels with lower gelatin content, longer PLA chains, and higher cross-linker concentrations (PEG–PLA1(1-Gel)9%, PEG–PLA2(1-Gel)9%, PEG–PLA3(1-Gel)9%, and PEG–PLA1(1-Gel)13%) were too stiff or degraded too slowly, which reduced their effectiveness for cell proliferate and function. The slow degradation rate may hinder the infiltration of surrounding nutrients and hinder the proper regeneration of cartilage tissue. Moreover, it could lead to the accumulation of waste products that negatively affect cell viability and functionality. Further studies of the specific hydrogel compositions for cartilage regeneration in vivo will be of great interest and benefit.

## 5 Conclusions

The PEG–PLA/gelatin hydrogel scaffold was successfully fabricated by the technique of 3D printing. The hydrogel composition affected its swelling ability and mechanical properties, which, in turn, influenced cell behaviors. An increase in the swelling ratio, caused by higher gelatin content or lower cross-linker concentration and shorter PLA chain length, diminished the compressive properties of the hydrogels. High-swelling hydrogels favored cell proliferation, but cell function was rather closely related to the modulus and degradation properties of the hydrogel. The expression of the cartilage-related matrix was more pronounced in the hydrogel with high gelatin content, which possessed a low modulus and a proper degradation rate. In this study, the composition of the PEG–PLA/gelatin hydrogel was optimized for a superior effect on chondrogenic redifferentiation, a promising strategy for cartilage tissue engineering.
